# Prehospital Intervention Among Black Patients With Traumatic Injury in Los Angeles County

**DOI:** 10.1001/jamanetworkopen.2024.36136

**Published:** 2024-09-27

**Authors:** Lindsey Loss, Martin Schreiber, Kazuhide Matsushima, Luis Tinoco-Garcia, Li Ding, Kenji Inaba, Reynold Henry

**Affiliations:** 1 Division of Trauma, Critical Care & Acute Care Surgery, Oregon Health & Science University, Portland; 2 Division of Acute Care Surgery, University of Southern California, Los Angeles; 3 Department of Preventive Medicine, University of Southern California, Los Angeles; 4 Division of Trauma, Emergency General Surgery and Critical Care Surgery, Department of Surgery, University of Nebraska Medical Center, Omaha

## Abstract

This cohort study examined the differences in prehospital treatment received by patients with traumatic injury belonging to different racial and ethnic groups between 2013 and 2020 in Los Angeles County.

## Introduction

Racial and ethnic disparities are well documented throughout the health care system.^1,2,3,4^ For example, in nontraumatic prehospital settings, patients are administered different levels of pain medication, which appears to be influenced by race.^3^ In traumatic settings, Black patients have repeatedly been shown to have worse outcomes and increased rates of mortality.^1,2^ However, the traumatic prehospital setting has not been well described regarding race and ethnicity. This study sought to identify racial disparities pertaining to prehospital interventions. Specifically, we aimed to examine differences with use of prehospital needle decompression because this intervention has been associated with a 25% decrease in 24-hour mortality.^5^ We hypothesized that Black patients would receive fewer prehospital interventions.

## Methods

We conducted a retrospective cohort study (2013-2020, analysis performed November 2023) using the Los Angeles County (LAC) Department of Health Services (DHS) Emergency Medical Services (EMS) database, which contains deidentified data. This study was approved by the institutional review board of the University of Southern California and followed the STROBE reporting guideline. Inclusion criteria were based on LAC Department of Health Services guidelines for prehospital needle thoracostomy (NT): decreased or absent breath sounds on 1 or both hemithoraces and systolic blood pressure (SBP) less than 90 mm Hg, traumatic pulseless electrical activity cardiac arrest, or trauma patients requiring positive pressure ventilation who develop hypoxia or severe hypotension. We excluded pediatric patients and those with missing data on race and ethnicity. They were divided into groups based on race and ethnicity as identified by EMS or self-identified by the patient. Univariate analyses were performed to compare demographics, injury type, EMS level, transportation time, and outcomes between groups. Hypothesis testing for categorical variables was performed using the χ^2^ or Fisher exact test as appropriate. With White patients as reference, multivariate logistic regression analysis was used to assess the association between racial identity and the primary intervention; placement of NT in field and secondary outcome interventions included airway adjunct use (oropharyngeal and nasopharyngeal airways) and bag-valve mask (BVM) use. Covariates used in the models included age, sex, injury mechanism, prehospital vitals, transportation type and time, severe injury (Abbreviated Injury Scale ≥3) in any body region, Injury Severity Score (ISS), and interaction between age and ISS. We considered *P* < .05 significant.

## Results

We identified 19 588 patients (Figure). Baseline differences were found between racial and ethnic groups, including age (mean [SD] age: Asian, 53.9 [21.3] years; Black, 40.4 [15.8] years; Hispanic, 40.3 [17.2] years; White, 45.1 [19.2] years; other, 45.8 [19.2] years; *P* < .001), percentage of male patients per racial group (Asian, 60%; Black, 78.8%; Hispanic, 79.4%; White, 74.7%; other, 74.7%; *P* < .001), initial blood pressure (mean [SD] SBP: Asian, 132 [35] mm Hg; Black, 124 [37] mm Hg; Hispanic, 126 [34] mm Hg; White, 129 [33] mm Hg; other, 130 [35] mm Hg; *P* < .001), initial heart rate (mean [SD] heart rate: Asian, 92 [24] beats per minute [bpm]; Black, 92 [28] bpm; Hispanic, 95 [26] bpm; White, 92 [24] bpm; other, 93 [25] bpm; *P* < .001), and percentage of patients with an initial Glasgow Coma Scale score of lower than 8 (Asian, 18% [216 of 1201]; Black, 22.7% [631 of 2777]; Hispanic, 18.9% [1385 of 7355]; White, 14.7% [888 of 5996]; other, 17.3% [137 of 789]; *P* < .001), percentage of patients with penetrating trauma (Asian, 8.7% [104 of 1201]; Black, 38.2% [1060 of 2777]; Hispanic, 23.4% [1723 of 7355]; White, 9.1% [544 of 5996]; other, 17.5% [138 of 789]; *P* < .001), and percentage of patients with an ISS higher than 25 (Asian, 32% [384 of 1201]; Black, 35.4% [984 of 277]; Hispanic, 31.4% [2313 of 7355]; White, 25.5% [1528 of 5996]; other, 29.2% [230 of 789]; *P* < .001). Our univariate analysis revealed Black patients received more NTs (Asian, 1.5% [18 of 1201]; Black, 5.2% [144 of 2777]; Hispanic, 3.8% [280 of 7355]; White, 1.9% [115 of 5996]; other, 2.5% [20 of 789]; *P* < .001). However, in multivariate analysis, there was a significant association of race and ethnicity with placement of NT and airway adjunct use, with Black patients receiving fewer NT and airway adjuncts (Table). There were no associations of race and ethnicity with BVM use.

**Figure.**
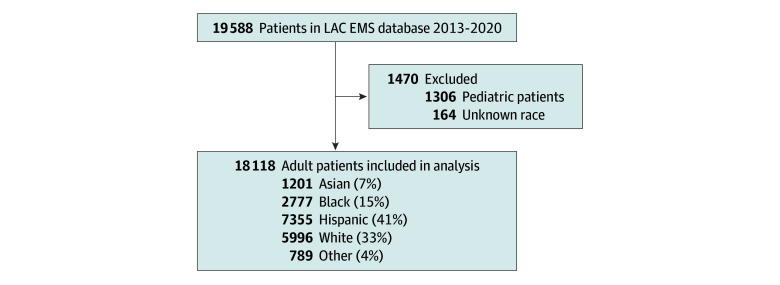
Patient Included in the Study LAC EMS indicates Los Angeles County Emergency Medical Services.

**Table.** Outcome Measures by Race and Ethnicity

Measure	Odds ratio (95% CI)	*P* value
Needle thoracostomy		
Asian	0.91 (0.38-2.16)	.82
Black	0.83 (0.67-0.92)	.04
Hispanic	1.49 (0.99-2.23)	.06
White	1 [Reference]	
Other^a^	1.09 (0.50-2.40)	.83
Bag-valve mask		
Asian	0.66 (0.38-1.12)	.12
Black	0.94 (0.63-1.40)	.76
Hispanic	0.88 (0.64-1.20)	.42
White	1 [Reference]	
Other^a^	1.23 (0.66-2.30)	.51
Airway adjunct use		
Asian	0.97 (0.53-1.79)	.94
Black	0.63 (0.40-0.88)	.04
Hispanic	0.95 (0.65-1.39)	.80
White	1 [Reference]	
Other^a^	0.58 (0.23-1.47)	.25

^a^ The “Other” category was coded by Emergency Medical Services (EMS) and included patients for whom EMS was not able to identify their race and ethnicity.

## Discussion

Black patients had a lower odds of NT placement and airway adjunct use in multivariate analysis. This discrepancy indicates the continued need for study and correction of racial and ethnic disparities. We are limited in our generalizability given the diversity of LAC as well as our use of an EMS-reported database leading to potential reporting errors. Understanding and addressing these disparities not only are a matter of social justice but are also crucial for fostering a health care system that truly prioritizes the well-being of every individual, regardless of their racial or ethnic background.

## Notes

Supplement.
Data Sharing Statement
