# Error in Figure

**DOI:** 10.1001/jamanetworkopen.2024.46920

**Published:** 2024-10-31

In the Research Letter titled “Prehospital Intervention Among Black Patients With Traumatic Injury in Los Angeles County,”^1^ published September 27, 2024, the “5996 White (33%)” and “789 Other (4%)” categories were either incorrectly labeled or missing from the list of “18 118 Adults patients included in the analysis.” This article has been corrected online.^1^
