# Supplementary material for: Prehospital Intervention Among Black Patients With Traumatic Injury in Los Angeles County
Source: JAMA Netw Open. 2024 Sep 27;7(9):e2436136. doi: 10.1001/jamanetworkopen.2024.36136 (PMC11437375; doi:10.1001/jamanetworkopen.2024.36136)
Supplement: Supplement. — Data Sharing Statement [file jamanetwopen-e2436136-s001.pdf]

Data Sharing Statement

Loss. Prehospital Intervention Among Black Patients With Traumatic Injury in Los Angeles County. *JAMA Netw Open*. Published September 27, 2024.
doi:10.1001/jamanetworkopen.2024.36136

Data
Data available: No

Additional Information
Explanation for why data not available: Data available via LA County EMS as needed
